# Personal factors influencing female students’ condom use at a higher education institution

**DOI:** 10.4102/phcfm.v16i1.4337

**Published:** 2024-02-19

**Authors:** Danelia McCarthy, Rehanna T. Felix, Talitha Crowley

**Affiliations:** 1 Department of Nursing and Midwifery, Faculty of Medicine and Health Sciences, Stellenbosch University, Cape Town, South Africa; 2 School of Nursing, Faculty of Community and Health Sciences, University of the Western Cape, Cape Town, South Africa

**Keywords:** condom utilisation, higher education institution, female students, personal factors, self-efficacy

## Abstract

**Background:**

South African female students’ consistent condom use is low, possibly due to personal factors, such as knowledge about sexual reproductive health, attitudes towards safe sex, risk perceptions and condom use, self-efficacy.

**Aim:**

This study aimed to investigate the personal factors that influence condom utilisation among female students.

**Setting:**

This study was conducted at a higher education institution in the Northern Cape province in South Africa.

**Methods:**

A quantitative, descriptive survey design was used. Three hundred and eighty five participants were selected using convenience sampling. The research instrument was a self-administered questionnaire, and the data were analysed using the Statistical Package for the Social Sciences, version 28.

**Results:**

Almost two-thirds (250, 64.9%) of participants used condoms to prevent pregnancy, sexually transmitted infections (STIs), and human immunodeficiency virus (HIV). Although attitudes towards safe sex were generally positive, low risk perceptions were reported. Consistent use of condoms was found in 32.2% (124) of participants, while 45.3% (174) participants used condoms inconsistently or never. A significant finding was that consistent use increased the likelihood of negotiating for a condom with partners by 9.14 times and confidence in putting one on for a partner by 8.05 times.

**Conclusion:**

The findings depict average levels of the use of condoms among female students. Prevention efforts should concentrate on educating female students to strengthen condom use and self-efficacy.

**Contribution:**

This study, supporting existing literature, suggests that preventative efforts should focus on educating young women about condom use, self-efficacy and encouraging STI conversations with sexual partners.

## Introduction

In sub-Saharan Africa, females between the ages of 15 and 19 years account for six out of seven new human immunodeficiency virus (HIV) infections, whereas females between the ages of 15 years and 24 years have twice the risk of contracting HIV.^1^ In the year 2022, 4 200 females between the ages of 15 years and 24 years were infected with HIV per week.^1^ As part of the strategy to improve sexual and reproductive health among young people, the South African Department of Social Development (DSD) launched the YOLO (You Only Live Once) programme. The programme targets young people aged between 15–24 years to address the social and behavioural causes of HIV. The programme aims to develop resilience in adolescents, addressing risk-taking pressures and integrating health and well-being, as a first step towards minimising HIV transmission and unintended pregnancies.^2^

Sexual reproductive health behaviours such as condom utilisation are complex and dependent on interrelated factors. Bandura’s theory depicts health behaviours as an interrelation between personal factors and the environment. Personal factors can include knowledge about sexual reproductive health, attitudes towards safe sex, risk perceptions and condom use self-efficacy.^3^

Although sexually active female students believe that knowledge about sexual reproductive health and contraception is vital, there are gaps in their knowledge.^4^ Furthermore, their misconceptions and the lack of awareness about condom utilisation led to high-risk behaviours.^5,6^ Female students’ sexual knowledge might influence their reproductive health behaviour and their sexual attitudes directly or indirectly.^7^

Most female students in Karnataka, India, have negative attitudes towards condom utilisation as they believe that condom utilisation will interfere with their sexual pleasure.^8^ In addition, students from Mahalapye, Botswana, believe they have the chance to live life to the fullest without limitations, hence they experiment with various partners without using condoms to better their sexual arousal.^9^ A study conducted among female university students in Durban, KwaZulu-Natal, South Africa, uncovered many characteristics that encourage and discourage female condom use. Protection from sexually transmitted diseases (including HIV and acquired immunodeficiency syndrome [AIDS]) and unintended pregnancy prevention made the device more appealing to female students. Furthermore, students had favourable opinions towards the female condom and preferred it to hormonal contraceptives because it provided them with dual protection.^10^

Sexual self-efficacy is centred on the person’s control over their sexual life^11^ which can be influenced by peer pressure^12^ and alcohol and substance abuse.^13^ Studies conducted in sub-Saharan Africa indicated that a greater degree of sexual self-efficacy was associated with increased safe-sex practice.^14^

### Problem statement

In South Africa, the disease burden from unprotected sexual encounters has significantly impacted young women between the ages of 15 years and 25 years. This resulted in an increase in intended births in the Northern Cape province from 6.6% to 20.9% between 1998 and 2016.^15^ A national study of 4500 first-year female Technical and Vocational Education and Training (TVET) students from 50 South African TVET colleges found that unintended pregnancy was common (74.6%) among the students.

Contrary to expectations, attending a higher education institution does not always result in greater contraception and sexual health awareness.^5^ As a primary health care (PHC) practitioner working at a campus clinic of a higher education institution, frequent diagnosis of STIs and unintended pregnancies among some young female students who do not use any kind of contraception was reported. The majority of these students come from underprivileged backgrounds and rural communities in the Northern Cape province. The Northern Cape province had a 26.4% poverty gap in 2011, which increased to 28.0% in 2015, and poor people in rural areas were significantly less fortunate than poor people in urban areas.^15^ This emphasises the urgency of investigating the personal factors influencing condom utilisation specifically among female students in this setting. Addressing these factors is crucial for developing targeted interventions aimed at promoting safe sexual practices and reducing the incidence of unintended pregnancies and STIs, particularly among the vulnerable demographic originating from underprivileged backgrounds and rural communities in the Northern Cape province.

### Research aim

This study aimed to investigate the personal factors that influence condom utilisation among female students.

## Research methods and design

### Study design

A quantitative, descriptive research design was used.^16^ Quantitative research was used to examine relationships among variables.^16^ Exploratory research is used to define problems, clarify concepts and develop hypotheses.^16^

### Study setting

There are various higher education institutions in the Northern Cape province of South Africa, including one university, a nursing college and a TVET with satellite campuses. The research was conducted at one of the higher education institutions in Kimberley in the Northern Cape province which has a student population of 2300. Most of the students studying at the selected higher education institution come from the larger Northern Cape province.

### Sampling and participants

The total population of the study was 877, of which a sample size of 385 was selected using convenience sampling until the minimum required sample size was reached. The minimum sample size was determined based on an estimated proportion of condom use of 50% (0.5) and a level of precision of ±5% (95% confidence interval).

Eligibility criteria included all female students aged 17–24 years studying at a higher education institution. As indicated in the literature, the age groups from 15 to 24 years are most at risk of acquiring HIV.^1^

### Data collection instrument and procedure

Following institutional approval, a discussion with a member of the Student Representative Council (SRC) and group administrators from several student WhatsApp groups was held. The information was passed on to the SRC and different administrators of student platforms via WhatsApp by the researcher and then to the students. The SRC sent out a message on both the SRC and the student Facebook pages. The researcher and research assistants had no access to the WhatsApp groups used by the various student leaders to communicate research sites and timings.

The data were collected using a self-administered questionnaire used in a previous study^17^ that assessed sexual and reproductive health risk behaviours among South African university students. The self-administered questionnaire was adapted by the researcher to determine the factors that influence condom utilisation among young female students at a higher education institution in the Northern Cape province.

The questionnaire consisted of four sections, namely, the background information of the participants (5 items), personal factors (21 items), environmental factors (8 items) and behaviour (12 items). For the purpose of this article, personal factors and behaviour are reported. The section on personal factors included questions on the: (1) knowledge about sexual and reproductive health (one multiple choice and six yes/no questions); (2) attitudes towards safe sex (five 5-point Likert scale questions); (3) risk perceptions (four 5-point Likert scale questions) and (4) condom use (Likert scale questions). Consistent condom use was measured by one 5-point Likert scale item asking participants how often they used condoms when having sex. The answer ‘always’ was considered consistent, while ‘most of the time’, ‘sometimes’, ‘rarely’ or ‘never using a condom/abstinent’ were considered inconsistent/no condom utilisation.

Data were collected in seven groups at prearranged venues on campus from 07 September 2021 to 10 October 2021. The researcher and two research assistants collected the data after written consent was obtained from the participants. Participants took an average of 30 min to complete the questionnaire.

### Validity and reliability

Content validity was ensured by basing the questionnaire on insights from the literature review, utilising an existing questionnaire^15^ and modifying it to align with the research framework. In addition, the questionnaire was reviewed by two experts, a psychologist and another primary health care nurse who works with young adults, to evaluate the content validity and face validity. Prior to the main study, on 06 September 2021, a pilot test with 39 participants was conducted to determine the reliability, readability and clarity of the questions. The pilot test was conducted in the library auditorium and was scheduled for 17:00. Although there were 45 students, not all of them completed the questionnaires because some chose not to participate in the pilot study. The pilot study data were not included in the main study. To assess reliability, Cronbach’s alpha internal consistency reliability test was calculated as 0.74 on the condom use self-efficacy scale as this was an established subscale.^18^

### Data analysis

Data were captured by the researcher in Microsoft Excel and analysed by a biostatistician using the Statistical Package for the Social Sciences version 28. Data were analysed descriptively using distributions. The chi-square test of independence and the odds ratio were used to describe the relationship between personal factors and behaviour. ‘Consistent’ condom utilisation was the dependent variable and the personal factors were the independent variables. Where the independent variables had more than two response categories, these were collapsed into binary/dichotomous or ‘yes’/‘no’ categories, to calculate odds ratios. A level of significance of *p* < 0.05 was used.

### Ethical considerations

The researcher obtained ethical approval from Stellenbosch University’s Health Research Ethics Committee (S21/03/048) and the higher education institution. Participation was voluntary and the participants gave written informed consent. The participants were informed that they could withdraw from the study at any time without consequences. The participants’ right to autonomy was observed. A psychologist was on standby should any participant need a referral; however, this was not needed.

## Results

The sample included 385 participants. The average age was 20.2 years (standard deviation [s.d.] 1.5). Most of the participants were single (282, 73.2%) and in their first year of study (244, 63.4%). Although there was a small percentage of gender nonconforming students (6, 1.6%), the participants were not excluded as it contributes to a more comprehensive understanding of the studied population and promotes inclusivity. The background information is indicated in Table 1.

**TABLE 1:** Background information of participants.

Variable	Frequency (*n*)	%
**Gender**		
Female	379	98.4
Nonconforming	6	1.6
**Marital status**		
Single	282	73.2
Married	5	1.3
In relationship	96	24.9
**Place of residence**		
On campus	347	63.4
At home with parents	4	1.0
Private residence	31	8.1
Family and friends	3	0.8
**Year of study**		
First	244	63.4
Second	80	20.8
Third	50	13.0
Fourth	11	2.9

### Knowledge about sexual and reproductive health

Most participants indicated that they used condoms for preventing pregnancy, STI and HIV (250, 64.9%), while others only used condoms for pregnancy prevention (24, 6.2%) or STI prevention (39, 10.1%). Seven (1.8%) of the study participants stated they do not use condoms and 65 (16.9%) stated they are not having sex or were abstinent.

Table 2 depicts the sexual reproductive health knowledge of participants. Areas where participants had low levels of knowledge (< 70%) were putting on or inserting a condom correctly for themselves (222, 58%) and their partner (192, 49.9%) and the different types of sexual intercourse (236, 61.3%).

**TABLE 2:** Sexual reproductive health knowledge.

Question	Yes, I know		No, I don’t know	
	*n*	%	*n*	%
I know what an unintended pregnancy is	328	85.2	57	14.8
I know how to put on or insert a condom correctly	222	58.0	162	42.0
I know how to insert or put on a condom for my partner(s)	192	49.9	193	50.1
I know the different types of sexual intercourse	236	61.3	149	38.7
I know where to get information on sexual and reproductive health	335	87.0	50	13.0
I know how to prevent pregnancy	366	95.1	19	4.9

### Attitudes towards safe sex

Overall, participants had positive attitudes towards safe sex (Table 3); however, 58.7% (226) of participants found it difficult to use a female condom and only 62.3% (240) felt that it is an excellent means of contraception. There was also uncertainty as to whether putting a condom on was their responsibility or their partner’s responsibility.

**TABLE 3:** Attitudes towards safe sex.

Question	Agree		Undecided		Disagree	
	*n*	%	*n*	%	*n*	%
I think it is important to use a condom every time I have penetrative sex	317	82.4	32	8.3	36	9.4
I think having safe sex is important for my future sexual and reproductive health	340	88.3	5	1.3	40	10.4
I feel that it is my partner’s responsibility to put a condom on	126	32.7	38	9.9	221	57.4
I find it difficult to use a female condom	226	58.7	104	27.0	55	14.3
I think that condoms are an excellent means of contraception	240	62.3	63	16.4	82	21.3

### Risk perceptions

Most participants perceived their HIV risk (310, 80.6%), STI risk (296, 76.9%) or pregnancy risk (306, 79.4%) as low. However, a quarter of participants (98, 25.4%) said it was unlikely they were the only person their partner has sex with (Table 4).

**TABLE 4:** Risk perceptions.

Question	Likely		Neutral		Unlikely		Sexually abstinent	
	*n*	%	*n*	%	*n*	%	*n*	%
What do you think is the likelihood for you to become pregnant in the next year?	19	5.0	60	15.6	306	79.4	0	0.0
Do you think you are the only person your partner(s) has sex with?	126	32.7	77	20.0	98	25.4	85	22.1
Do you feel you are at risk of becoming HIV positive?	29	7.6	46	11.9	310	80.6	0	0.0
Do you think you are at risk of getting a sexually transmitted infection (STI)?	40	10.4	49	12.7	296	76.9	0	0.0

### Condom use self-efficacy

As illustrated in Table 5, most participants (252, 65.4%) reported that they can refuse sex if their partner(s) refuse to use a condom; however, 185 (50%) reported they do not always carry a condom with them in case they need one. Only 153 (39.7%) of the 385 study participants reported they are confident to put a condom on for their partner(s).

**TABLE 5:** Condom use self-efficacy.

Question	Agree		Undecided		Disagree		Abstinence	
	*n*	%	*n*	%	*n*	%	*n*	%
I am confident to put a condom on for my partner(s)	153	39.7	58	15.1	92	23.9	82	21.3
I can negotiate condom use with my partner(s)	224	58.2	23	6.0	56	14.5	82	21.3
I can delay penetrative sex if a condom is not available	224	58.0	35	9.0	45	12.0	81	21.0
I can say no to having sex if my partner refuses to use a condom	252	65.4	27	6.8	29	7.6	77	20.0
I always carry a condom in case I might need one	133	33.0	67	17.0	185	50.0	N/A	N/A

### Condom use

Regarding consistent condom use, almost a third of participants (124, 32.2%) reported consistent condom use, 43.7% (168) reported inconsistent condom use and 6 (1.6%) reported never using a condom when having sexual encounters. Some participants (87, 22.6%) reported they are sexually abstinent.

Table 6 illustrates the personal factors significantly associated with consistent condom use. Study participants were 1.08 times more likely to know how to properly put on a condom if they used one consistently. Participants who engaged in regular condom usage exhibited significantly higher levels of confidence in putting on a condom for their partner(s) and in negotiating condom use with their partner(s), with an increase of 8.05 and 9.14 times, respectively. Moreover, those who consistently used condoms in the study were found to be 12.86 times more inclined to delay penetrative sex if a condom was unavailable and were 10.40 times more inclined to refuse sex if a condom was not available.

**TABLE 6:** Personal factors associated with consistent condom use.

Variable	Chi-square *p*-value	Odds ratio (OR)	95% Confidence interval (CI)
Knowledge: I know how to put on a condom correctly	0.016	1.08	1.05 – 1.1
Self-efficacy: I am confident to put a condom on for my partner(s)	< 0.01	8.05	3.1 – 18.1
Self-efficacy: I can negotiate condom use with my partner(s)	< 0.01	9.14	4.8 – 17.4
Self-efficacy: I can delay penetrative sex if a condom is not available	< 0.01	12.86	6.3 – 26.1
Self-efficacy: I can say no to having sex if my partner refuses to use a condom	< 0.01	10.40	5.6 – 19.2

## Discussion

The majority of participants (64.9%) in the study reported using condoms to prevent pregnancy, STIs and HIV, and 95.1% reported knowing how to prevent pregnancy. Similarly, in a study done at Nairobi’s Kirinyaga University, the majority of participants (93.6%) were aware that condoms provide protection against HIV and AIDS and unintended pregnancy.^19^ However, a study among sexually active female students in China found that while information regarding sexual reproductive health and contraception is important, there are gaps in their understanding.^4^ Knowledge is important as a lack of knowledge about condom use may result in high-risk behaviour,^5^ which may influence their reproductive health behaviour and sexual attitudes, either directly or indirectly.^7^ The relatively high self-reported knowledge level in this study may be attributed to comprehensive sex education delivered in public schools, although it would be difficult to measure the exposure of participants to this.^20^ Life Orientation sexuality education in schools addresses sexuality education, messages about preserving virginity before marriage, refraining from sex and using condoms when having sex.^21^

Regarding attitudes, the majority of research participants (88.3%) agreed that having safe sex is vital for their future sexual and reproductive health, and 82.4% indicated that using a condom every time they engage in sexual activity is crucial. This indicates positive attitudes towards safe sex and consistent condom use. A favourable attitude towards the usage of condoms may influence actual condom use behaviours. These findings are consistent with a previous study by Mbele et al., which found that female college students had a positive attitude towards the use of condoms to avoid STIs, unintended pregnancy and HIV and AIDS.^5^ On the other hand, a US study reported that although some female college students believed that using condoms would assist them in preventing STIs, HIV and AIDS and unintended pregnancy, condom use was still opposed by a large number of minority of students.^22^ This means that there may be subgroups with poor attitudes towards condom use that need to be addressed.

Attitudes and the perception of HIV risk were not associated with consistently using a condom. Participants in this study who use condoms consistently are 1.08 times more likely to know how to correctly use one. Conversely, a study of college students at a Portuguese university found no significant association between knowledge and consistent condom use, but a positive association between attitudes and consistent condom use.^23^ Previous research found that, despite increased prevention awareness among university students, the rate of inconsistent condom use was 83.5%, indicating either a lack of knowledge about reproductive health issues such as HIV and AIDS, STIs and unintended pregnancies, and the consequences of these or that other factors may influence behaviour.^13^

Despite overall good attitudes towards condom use, more than half of the participants (58.7%) reported difficulty using a female condom. In a study conducted in Karnataka, India, most female students had negative attitudes towards condom utilisation as they believed that condom utilisation would interfere with their sexual pleasure.^8^ A study of South African university and TVET college students indicated that the majority of participants found female condoms less appealing and more difficult to use than male condoms.^5^ This could mean that education on female condom use should be intensified.

The majority of participants (80.6%) considered their risk of obtaining HIV to be low, as well as their chance of contracting an STI (76.9%). According to previous research in Britain, only 2.5% of women perceived themselves to be highly at risk of HIV based on their current sexual lifestyle (high-risk perception), and a majority (86%) of those with high HIV risk perception had not had an HIV test in the previous year.^24^ It should be noted that the participants’ risk perceptions may not be a genuine reflection of their participants’ real risk. This could be due to participants in the study underestimating their risk of contracting STIs and/or HIV.^25^

In this study, although self-efficacy in using condoms ranged from 39.7% to 65.4%, putting on a condom for one’s partner had the lowest level of self-efficacy (39.5%). The levels of self-efficacy in condom negotiation reported in this study (58%) are slightly higher than those reported in a study conducted among female university students in serious relationships in KwaZulu-Natal, where 40% indicated that they could negotiate condom usage with their partners.^26^ Similarly, a study conducted in a Nigerian institution discovered low levels of self-efficacy in condom use. Despite knowing the risks, many female students were unable to negotiate for safer sex.^27^

While self-efficacy for condom use is higher in this study, as opposed to previous studies, it still needs to be improved. Even though it can be argued that the participants in the study who reported being sexually abstinent do not need to have condom use self-efficacy, they could benefit from increased sexual self-efficacy in order to be ready for a sexual relationship. Knowing how to put a condom on correctly and condom use self-efficacy were the only factors significantly associated with consistent condom use behaviour.

Participants in this study who used condoms regularly were 8.05–12.86 times more likely to exhibit condom use self-efficacy. Consistent with this, a study conducted in a minority-serving institution in the United States discovered that those who reported being able to put on or have their partner(s) put on a condom without ruining the mood were 7.7 times more likely to be a consistent condom user.^28^ Previous research discovered that self-efficacy for condom negotiation and preparatory safer sexual behaviour were significantly associated with consistent condom usage, as was abstinence, implying that women who exhibit this behaviour are more likely to use condoms.^29^

As condom use self-efficacy was associated with consistent condom use, higher education institutions should introduce programmes and activities that promote increased self-efficacy, self-esteem and sexual reproductive health and well-being in young women. One way to address a lack of self-efficacy is through education, for example, the YOLO programme. As the programme aims to develop resilience in adolescents to cope with risk-taking, it may have an impact on their self-efficacy.^2^ However, the impact of the YOLO programme on young people between the ages of 15 and 24 years needs to be evaluated, in order to extend the education and training to female students in higher education institutions.

### Study limitations

The limitation of the study was that participants might not have reported their opinions or practices authentically. Further, convenience sampling meant participants self-selected to participate. The findings may not be generalisable to other higher education institutions.

## Conclusion

On average, female students at a higher education institution in the Northern Cape Province reported using condoms. Condom use self-efficacy was associated with consistent condom utilisation rather than condom knowledge, suggesting that prevention efforts should focus on educating young females to increase condom use self-efficacy and to encourage sexually transmitted infections conversations with sexual partners.
